# Risk Factors for Reintervention With Functionally Single-Ventricle Disease Undergoing Staged Palliation in England and Wales: A Retrospective Cohort Study

**DOI:** 10.1161/CIRCULATIONAHA.123.065647

**Published:** 2023-10-24

**Authors:** Qi Huang, Deborah Ridout, Victor Tsang, Nigel E. Drury, Timothy J. Jones, Hannah Bellsham-Revell, Elena Hadjicosta, Anna N. Seale, Chetan Mehta, Christina Pagel, Sonya Crowe, Ferran Espuny-Pujol, Rodney C.G. Franklin, Kate L. Brown

**Affiliations:** Clinical Operational Research Unit, Department of Mathematics (Q.H., E.H., C.P., S.C., F.E.-P.; Population, Policy and Practice Programme, Great Ormond Street Institute of Child Health (D.R.), University College London.; Institute of Cardiovascular Science (V.T., K.L.B.), University College London.; Great Ormond Street Hospital Biomedical Research Centre, London (V.T., K.L,B,).; Paediatric Cardiology and Cardiac Surgery, Birmingham Children’s Hospital, Birmingham (N.E.D., T.J.J., A.N.S., C.M.).; Institute of Cardiovascular Sciences, University of Birmingham (N.E.D., T.J.J., A.N.S.).; Paediatric Cardiology, Evelina London Children’s Hospital (H.B.-R.).; Paediatric Cardiology, Royal Brompton and Harefield NHS Foundation Trust, London (R.C.G.F.).

**Keywords:** cardiac surgical procedures, heart defects, congenital, risk factors

Our study aimed to evaluate population-based rates of, and risk factors for, cardiac reinterventions in children with functionally single-ventricle (f-SV) congenital heart disease. A retrospective cohort study was undertaken, including all children born in England and Wales with f-SV congenital heart disease^1^ between 2000 and 2018 who underwent any initial or staged palliative procedures.^1,2^ The National Congenital Heart Diseases Audit (registry) was used, with National Health Service Research Ethics Committee approval; the study dataset is available only on this basis. Five-year survival, as ascertained in 2020, was 72.1% (95% CI, 70.6%–73.7%).^1^ The study outcome was any cardiac operation or interventional catheter undertaken in addition to the planned treatment pathway. The association between prespecified risk factors (see Table) and the cumulative incidence of additional procedures was investigated using multivariable Fine-Gray regression. Competing events were death and next staged treatment completion or heart transplant without additional procedures.

**Table.**
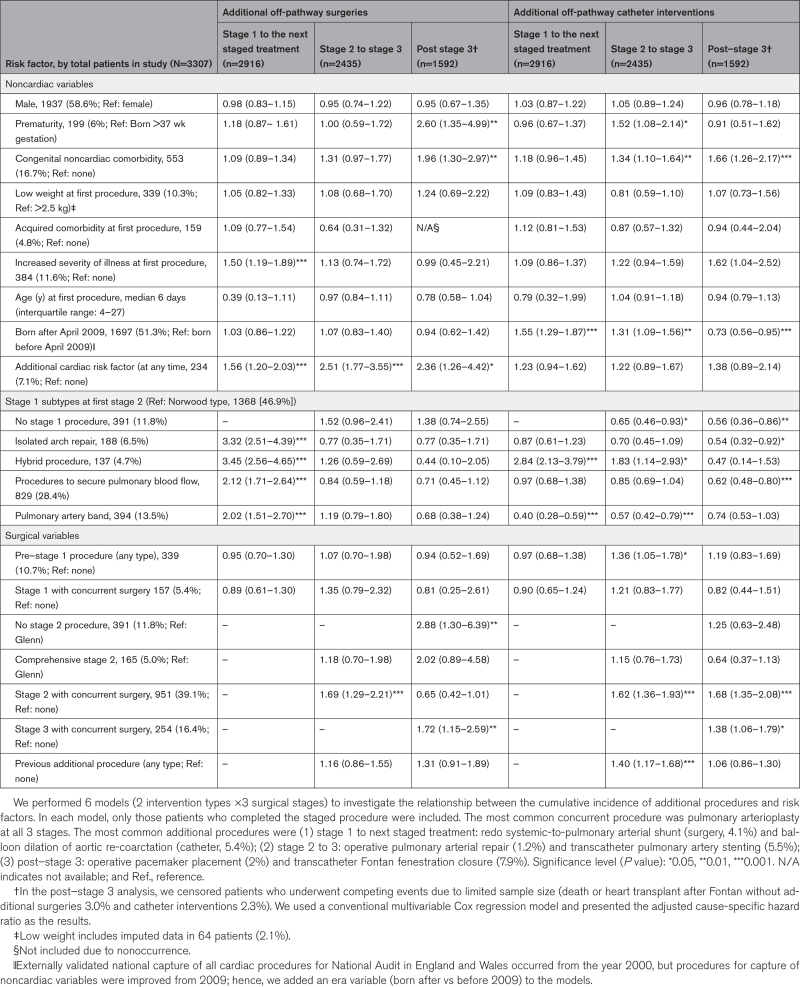
Adjusted Subdistribution Hazard Ratios (With 95% CIs) for the Occurrence of Additional Procedures in Children With Functionally Single-Ventricle Disease at the 3 Stages of Interventional Treatment

Among 3307 children with f-SV there were 1266 (38.3%) children with hypoplastic left heart syndrome; 448 (13.6%) with tricuspid atresia; 328 (9.9%) with double-inlet left ventricle; 243 (7.4%) with f-SV with atrial isomerism; 231 (7.0%) with unbalanced atrioventricular septal defect; 138 (4.2%) with pulmonary atresia; 112 (3.4%) with mitral atresia without hypoplastic left heart syndrome; and 541 (16.4%) with other f-SV. Median first procedure age was 6 days (interquartile range [first and third quartiles], 4–27); at this timepoint 384 (11.6%) had increased severity of illness. Congenital comorbidity was present in 553 (16.7%) patients, and 199 (6.0%) had premature birth.

We identified the following cardiac procedures as on the planned treatment pathway^2^ (not the study outcome): interventions before stage 1 in 339 (10.3%) patients (eg, balloon atrial septostomy 195 [5.8%]); first stage 1 procedure in 2916 (88.2%) patients (subtypes in Table); second stage in 2435 (73.6%) patients (Glenn 2270 [68.6%]) and comprehensive stage 2 operation in 165 (5.0%); third stage (Fontan-type) operation in 1592 (48.1%) patients; and heart transplant in 47 (1.4%) patients.

Over a median follow-up of 5.4 years (interquartile range [first and third quartiles], 0.8–10.8) of 3307 patients, 1730 (52.3%) patients had at least 1 additional procedure (ie, the study outcome), and 887 (26.8%) patients had multiple additional procedures. Of 3427 additional procedures, 1289 (37.4%) were cardiac surgery in 921 patients, and 2138 (62.4%) were interventional catheters in 1293 patients. In the Table, we show the adjusted subdistribution hazard ratios with each risk factor for additional procedures, from the first stage 1 procedure to the next stage, whichever stage occurred next (additional surgery in 596 [20.4%], and catheter intervention in 596 [20.4%] patients); from stage 2 to 3 (265 [10.9%] and 616 [25.3%]); and after stage 3 (135 [8.5%] and 387 [24.3%]).

Between stage 1 and the next stage that occurred, all stage 1 subtypes were associated with higher risk of additional surgery, compared with the reference category of Norwood, most notably hybrid (adjusted subdistribution hazard ratio, 3.45 [95% CI, 2.56–4.65]; *P*<0.001). The hybrid was also associated with higher risk of additional catheter intervention after stage 1 (2.84 [2.13–3.79]; *P*<0.001); and between stages 2 and 3 (1.83 [1.14–2.93]; *P*<0.05). Nonhybrid stage 1 subtypes were all associated with lower risk than the Norwood, of additional catheter procedures at later stages.

Increased severity of illness (ie, ventilation, shock) before the first procedure was associated with higher risk of additional surgery after stage 1 (1.50 [1.19–1.89]; *P*<0.001).

When children underwent more interventions than the 3 planned palliative stages, subsequent additional procedures were more likely, as shown by increased risk of additional catheter interventions between stages 2 and 3, after a pre–stage 1 procedure (1.36 [1.05–1.78]; *P*<0.05); or after an additional procedure between stages 1 and 2 (1.40 [1.17–1.68]; *P*<0.001). Concurrent surgery with stage 2, was associated with increased risk for additional surgery (1.69 [1.29–2.21]; *P*<0.001), and additional catheters between stages 2 and 3 (1.62 [1.36–1.93]; *P*<0.001); and after stage 3 (1.68 [1.35–2.08]; *P*<0.001). Concurrent surgery with stage 3 was associated with higher risk of subsequent additional surgery (1.72 [1.15–2.59]; *P*<0.01) and catheter intervention (1.38 [1.06–1.79]; *P*<0.05).

Children with complex features^3^ were overrepresented; with acquired cardiac risk factors (eg, impaired ventricular function, raised pulmonary vascular resistance), there was increased risk of additional surgery after stage 1 (1.56 [1.20–2.03]; *P*<0.001), between stages 2 and 3 (2.51 [1.77–3.55]; *P*<0.001), and after stage 3 (2.36 [1.26–4.42]; *P*<0.05). With congenital comorbidity, there was increased risk of additional catheter interventions after stage 2 (1.34 [1.10–1.64]; *P*<0.01) and stage 3 (1.66 [1.26–2.17]; *P*<0.001); and additional surgery after stage 3 (1.96 [1.30–2.97]; *P*<0.01). With premature birth, there was increased risk after stage 3 of additional surgery (2.60 [1.35–4.99]; *P*<0.01) and after stage 2 catheter intervention (1.52 [1.08–2.14]; *P*<0.05).

Parents and clinicians should be prepared for additional procedures in the early years for most children with f-SV disease. Although it is infeasible to adjust for all aspects of case complexity, additional procedures could represent a disadvantage of the hybrid pathway. As previously reported,^4,5^ the pulmonary arteries most often require additional interventions in f-SV, especially transcatheter beyond stage 2. Strategies to minimize pulmonary arterial distortion, preserve ventricular function, and maintain lower pulmonary vascular resistance may protect children from additional procedures during childhood.

## ARTICLE INFORMATION

### Sources of Funding

This study was funded by the British Heart Foundation (Project Grant No. PG/17/88/33401). Drs Tsang and Brown received support from the NIHR Biomedical Research Centre at Great Ormond Street Hospital.

### Disclosures

The study was approved by the UK National Health Service Stanmore Research Ethics Committee (Reference 18/LO/1688) and the need for patient consent was waived.
